# Lyme Disease Biobank: 10 years of 3 month follow-up visits from 2014 to 2023

**DOI:** 10.3389/fmed.2025.1577936

**Published:** 2025-07-10

**Authors:** Elizabeth J. Horn, George Dempsey, Anna M. Schotthoefer, Matthew McArdle, Allison F. Weber, Cathy De Luca, Bobbi S. Pritt, Elizabeth L. Maloney

**Affiliations:** ^1^Lyme Disease Biobank, Portland, OR, United States; ^2^East Hampton Family Medicine, East Hampton, NY, United States; ^3^Marshfield Clinic Research Institute, Marshfield, WI, United States; ^4^Department of Pathology, Stony Brook University, Stony Brook, NY, United States; ^5^Mayo Clinic, Rochester, MN, United States; ^6^Partnership for Tick-borne Disease Education, Wyoming, MN, United States

**Keywords:** Lyme Disease, serology, seroconversion, biorepository, Biobank, antibiotic treatment

## Abstract

**Introduction:**

Lyme Disease Biobank (LDB) enrolls participants with signs and symptoms of early Lyme disease (LD) from endemic areas and makes samples available to researchers developing more accurate diagnostics. From 2014 to 23, 466 cases and 367 controls were enrolled on Long Island, NY, and in Central Wisconsin.

**Methods:**

This study included 253 LDB participants who provided samples from an initial and a convalescent blood draw. Serologic testing, including a first-tier enzyme immunoassay and IgM and IgG immunoblotting, was performed on all samples; blots were interpreted using CDC criteria.

**Results:**

At the first draw, 34% of samples from participants presenting with erythema migrans (EM) > 5 cm were positive by CDC’s standard two-tiered testing (STTT) algorithm. IgG seroconversion was rare, only 4% of samples demonstrated seroconversion. While the majority of participants (78%) reported no LD symptoms at the second draw, 22% reported ongoing symptoms; the most common being joint pain, fatigue, and muscle pain. Only 35% of participants with ongoing symptoms reported seeing their provider about their symptoms.

**Conclusion:**

These results provide additional evidence that STTT is insensitive in early LD and seroconversion is rare after antibiotics. More than one-fifth of participants initially prescribed antibiotics reported ongoing LD symptoms. Therefore, healthcare professionals treating patients with early LD are encouraged to follow-up with their patients, determine whether they continue to experience symptoms, and consider immediate antibiotic re-treatment as appropriate. Early diagnosis, treatment, and follow-up of early LD patients has the potential to improve outcomes and reduce the burden of LD in the US.

## Summary

Standard two-tiered testing is insensitive in early Lyme disease, and seroconversion is rare after antibiotic treatment. More than one-fifth of participants initially prescribed antibiotics experienced ongoing symptoms that they attributed to Lyme disease ∼3 months after treatment.

## Introduction

Lyme Disease (LD), the most common vector-borne disease in the United States, is primarily caused by infection with the bacteria *Borrelia burgdorferi sensu stricto* and, rarely, *Borrelia mayonii.* The pathogen is transmitted through the bite of an infected Ixodes tick (1, 2). Using insurance claims data, CDC has estimated that ∼476,000 people or more are diagnosed with LD each year (3). Early LD symptoms within 30 days of infection are non-specific and include viral illness-like symptoms such as headache, fatigue, body aches, joint pain, and fever (1, 4). Erythema migrans (EM), an annular, erythematous, expanding skin lesion may be present in early LD (1, 4). As the infection progresses, *Borrelia* can disseminate throughout the body, impacting the joints, nervous system, and heart (1, 4, 5).

Early diagnosis and adequate antibiotic treatment of LD are important strategies for improving outcomes. Early LD is generally diagnosed clinically by the presence of an EM of at least 5 cm (6) and may be supported by laboratory testing. EMs have multiple presentations, and while often described as a “bulls-eye” rash due to central clearing, this presentation is uncommon; the most common appearance is a red or pink (erythematous), homogenously colored annular lesion (7, 8). For patients presenting with EM in endemic areas, antibiotics are prescribed based on clinical diagnosis and laboratory testing is not recommended (9–11). For those presenting without EM, diagnosis is challenging.

Delays in diagnosis can make LD more difficult to treat, with longer durations between symptom onset and treatment leading to poorer outcomes (12). Treatment recommendations for uncomplicated early LD in the US is a course of antibiotics ranging from 10 to 28 days in duration (10, 13, 14). Untreated or inadequately treated early LD can result in disease progression, typically neurologic involvement or Lyme arthritis (15, 16).

Current laboratory testing for LD uses serology, an indirect test that measures the immune system’s humoral response to *Borrelia*. PCR of blood is not recommended as *Borrelia* are found only transiently in blood and migrate to other tissues (17). The standard two-tiered testing (STTT) algorithm includes a first-tier enzyme immunoassay (EIA); samples with positive or equivocal results undergo second-tier testing with IgM and IgG immunoblotting (9, 11, 18). More recently, modified two-tiered testing (MTTT) that replaces the confirmatory immunoblots with a second-tier EIA targeting a different *Borrelia* epitope than the first-tier EIA is being used (19). While MTTT has improved sensitivity over STTT (11), both STTT and MTTT remain insensitive in early LD (17).

When identified early and treated with antibiotics, most patients with LD recover from the infection and return to their pre-Lyme health status. However, ∼10–20% of people with LD who were treated go on to have ongoing, persistent symptoms (20). The most common persistent or relapsing symptoms following treatment include severe fatigue, cognitive issues, and musculoskeletal pain (21). Additionally, delays in treatment are associated with more persistent symptoms, and those presenting without EM are more likely to experience delays in diagnosis, and subsequent treatment delays (12). While the prevalence of people experiencing persistent LD symptoms is unknown, a 2019 analysis estimated that the prevalence of post-treatment LD would be between 1.6 million and 2.3 million cases in 2020 (22).

Lyme Disease Biobank (LDB) was created to provide well-characterized, real-world early LD samples to investigators developing more accurate diagnostics for LD and other tick-borne infections (TBI) (23). As part of sample characterization, LDB had serologic testing including a first-tier EIA and IgM and IgG immunoblotting performed on all samples collected. Here we evaluate serologic testing results from LDB participant samples provided at enrollment and ∼3 months later during the 10-year period from 2014 to 2023. Many of these participants, particularly those presenting with EM, would not have had LD testing as part of standard clinical care at the first visit and very few would have had a follow-up visit that included LD testing. This analysis also explores whether participants who returned for a second draw reported symptoms of LD similar to those at enrollment, if they saw their provider when symptoms persisted, and if additional courses of antibiotics were prescribed between the first and second visits when symptoms persisted.

## Materials and methods

Participants were enrolled in LDB with signs and symptoms of early LD as previously described (23). Briefly, sites were selected based on their location in Lyme-endemic areas and their ability to identify and enroll early LD patients. Enrollment criteria for cases included patients presenting with an EM or an erythematous annular, expanding lesion with or without symptoms, and patients presenting with viral-like symptoms and suspected tick exposure or tick bite but without an EM/annular lesion. For those presenting with EM/annular lesion, sites prioritized enrolling patients presenting with EM > 5 cm; there was no lower limit on size. Controls were identified as healthy individuals from the same areas without a history of LD or TBI. Participants from East Hampton (EH) and Wisconsin (WI) were enrolled under Advarra IRB protocol Pro00012408 and Marshfield Clinic Research Institute IRB protocol SCH20216, respectively. Enrollment criteria are summarized below.

**Table:** 

Enrollment type	Inclusion criteria	Exclusion criteria
Enrolled with EM	• Physician identification	• Immunocompromised
	• EM or annular expanding lesion	• < 10 yr of age
		• Antibiotics initiated > 48 h
		• Tick bite reaction only
Enrolled without EM	• Physician identification	• Immunocompromised
	• At least one of the following: headache, fatigue, fever, chills, joint pain, or muscular pain	• < 10 year of age
	• Suspected tick exposure/tick bite	• Antibiotics initiated > 48 h
		• History of chronic fatigue syndrome, rheumatologic disease, multiple sclerosis
Endemic controls	• Generally healthy individuals	• Immunocompromised
		• < 10 year of age
		• History of LD or TBI

An initial acute blood draw (first draw) was taken on enrollment, typically the same day as the participant was identified by the provider, and an optional convalescent blood draw (second draw) was taken 2–3 months later from participants who agreed to provide a second draw. Participants were not eligible if they had taken antibiotics for > 48 h at the time of enrollment. This analysis included only the laboratory testing results and survey responses of the 253 participants enrolled between 2014 and 2023 who provided both initial and convalescent blood samples.

Clinical data were collected using case report forms (CRFs) at both blood draws. Data collected at the initial draw included demographics, information about signs and symptoms of early LD, previous history of LD, and whether antibiotics were prescribed. Data collected at the convalescent draw included information about current LD symptoms, if they saw their provider about their symptoms, and if additional antibiotics were prescribed since enrollment. At the first draw, participants were asked which, if any of the following symptoms were present: fever, chills, fatigue, night sweats, nausea, headache, body aches, joint point, and neuralgia. Similarly, at the second draw, participants were asked which, if any, of the following symptoms were present: fatigue, night sweats, flu like symptoms, muscle pain, joint pain, cardiac/respiratory problems, gastrointestinal problems, confusion/memory loss, numbness/tremors, facial paralysis, vision problems, and hearing problems.

Blinded testing was performed at Stony Brook University (SB) and at Mayo Clinic (MC) as described (23) and summarized in the table below. First-tier EIAs and second-tier immunoblots were performed on all first and second draw samples at the end of each collection season for research purposes and not clinical care. All immunoblots were interpreted using CDC criteria. Testing at SB used a laboratory-developed ELISA based on whole cell lysate from *B. burgdorferi* and anti-*B. burgdorferi* IgM and IgG immunoblots. SB also performed C6 peptide ELISA (Oxford Immunotec, Malrborough, MA) testing on first and second draw samples until C6 was discontinued. MC used the following assays: for the first tier, C6 peptide ELISA was used in 2016 and VlsE/pepC10 IgM/IgG ELISA (Zeus Scientific, Raritan, NJ) was used in all other years; for the second tier, IgM and IgG immunoblotting was performed using ViraStripe blots or ViraChip assay (Viramed; Biotech AG, Germany).

STTT results at the first draw are available for 252 participants, as one participant only provided a whole blood sample and not a serum sample. Convalescent serologies are available for all 253 participants. C6 peptide ELISA was run in addition to the first-tier ELISA when it was available, and results from two ELISAs are available at the first and second draw for 107 participants enrolled at EH (2014–2020) and 79 participants enrolled at WI (2017–2020). SB and MC clinical laboratories are College of American Pathologists (CAP)-accredited and Clinical Laboratory Improvement Amendments (CLIA)-certified with experience in Lyme disease testing. No inter-laboratory comparisons were performed between the two clinical labs.

**Table:** 

Serology testing summary	EH	WI
Stony Brook (SB): Laboratory-developed ELISA and IgM and IgG Immunoblots	2014–2017	
Stony Brook (SB): C6 Peptide ELISA	2014–2020	2017–2020
Mayo Clinic (MC): C6 Peptide ELISA and IgM and IgG Immunoblots		2016
Mayo Clinic (MC): VlsE/pepC10 IgM/IgG ELISA and IgM and IgG Immunoblots	2018–2023	2017–2023

Cases were classified as Laboratory Confirmed LD, Probable LD, Suspected LD, and Symptomatic No Lesion (SNL) as previously described (23) and summarized in the table below.

**Table:** 

Case classification	Criteria
Laboratory Confirmed LD	Positive STTT result with or without EM OR 2 positive ELISAs with EM > 5 cm OR positive *Borrelia* PCR result OR IgG seroconversion
Probable LD	EM > 5 cm without confirmatory laboratory evidence
Suspected LD	EM < 5 cm without confirmatory laboratory evidence
SNL (Symptomatic No Lesion)	Symptomatic without EM and without confirmatory laboratory evidence

## Results

### Enrollment criteria and demographics

A total of 466 cases with signs and symptoms of early Lyme were enrolled in LDB from 2014 to 2023 (Table 1). Of these cases, nearly two-thirds (297, 64%) presented with an erythematous, annular, expanding skin lesion suspected to be EM, including 230 participants that had an EM > 5 cm. For those presenting with a suspected EM < 5 cm, 58 had a minimum diameter of 2 cm. Of the 466 cases, more than half (54%) returned for a second draw, occurring a median of 89 days (mean 98 days) after the first draw. All 253 participants who completed first and second draws were included in this analysis.

**TABLE 1:** Enrollment and symptom information for the current analysis.

Enrollment	Presenting w/EM > 5 cm (%)	Presenting w/EM < 5 cm (%)	Presenting w/o EM (%)	Total cases (%)
**Initial (1st) draw**	***N* = 230**	***N* = 67**	***N* = 169**	***N* = 466**
East Hampton (EH)	153 (67)	32 (48)	99 (59)	284 (61)
Wisconsin (WI)	77 (33)	35 (52)	70 (41)	182 (39)
**Convalescent (2nd) draw**	***N* = 128**	***N* = 39**	***N* = 86**	***N* = 253**
East Hampton (EH)	74 (58)	12 (31)	37 (43)	123 (49)
Wisconsin (WI)	54 (42)	27 (69)	49 (57)	130 (51)
**Symptoms at Enrollment for Participants with 2nd Draw**	*N* = 128* *N* = 126	*N* = 39* *N* = 38	*N* = 86* *N* = 81	*N* = 253* *N* = 245
Fatigue*	64 (50)	16 (41)	75 (87)	155 (61)
Body aches	64 (51)	13 (34)	68 (84)	145 (59)
Headache*	53 (41)	10 (26)	65 (76)	128 (51)
Joint pain	50 (40)	11 (29)	61 (75)	122 (50)
Chills	47 (37)	10 (26)	47 (58)	104 (42)
Night sweats	42 (33)	8 (21)	47 (58)	97 (40)
Fever*	27 (21)	7 (18)	47 (55)	81 (32)
Nausea	30 (24)	3 (8)	29 (36)	62 (25)
Neuralgia	24 (19)	5 (13)	24 (30)	53 (22)
No symptoms (EM only)	44 (34)	19 (50)	0 (0)	63 (25)

EM, erythema migrans; EH, East Hampton, NY; WI, Marshfield, WI, which includes samples from Lake Hallie, Marshfield, Minocqua, Rice Lake, Wausau, and Weston.

*Fatigue, headache, and fever were included on the CRF in 2014. Other symptoms (body aches, joint pain, chills, night sweats, nausea, and neuralgia) were added to the CRF in 2015. This is reflected in the denominators.

For these 253 participants, 75% reported at least 1 symptom at enrollment. The most commonly reported symptoms were fatigue (61%), body aches (59%), headache (51%), and joint pain (50%) (Table 1). All 86 participants without EM and 104 of the 167 (62%) participants with suspected EM reported symptoms of early LD (Table 1). Participant demographics are presented in Table 2. The average age of participants was 57; more men (58%) were enrolled than women (42%). Age and sex were consistent across both sites. Eighty-seven percent of the participants reported their race as white (87%); all of the 29 Hispanic or Latino participants were enrolled at EH.

**TABLE 2:** Demographic information of participants returning for second draw.

Demographic information	East Hampton *N* = 123	Wisconsin *N* = 130	Total *N* = 253
**Age**			
Median (mean) age	56 (55)	62 (59)	59 (57)
Age (range)	(15-90)	(11-93)	(11-93)
** Sex**			
Female (%)	53 (43)	52 (40)	105 (42)
Male (%)	70 (57)	78 (60)	148 (58)
** Race**			
Asian (%)	1 (1)	1 (1)	2 (1)
Black (%)	1 (1)	0 (0)	1 (1)
Hispanic or Latino (%)	29 (24)	0 (0)	29 (11)
White (%)	92 (75)	127 (98)	219 (87)
Mixed race (%)	0 (0)	2 (2)	2 (1)

### LDB sample characterization

**FIGURE 1:**
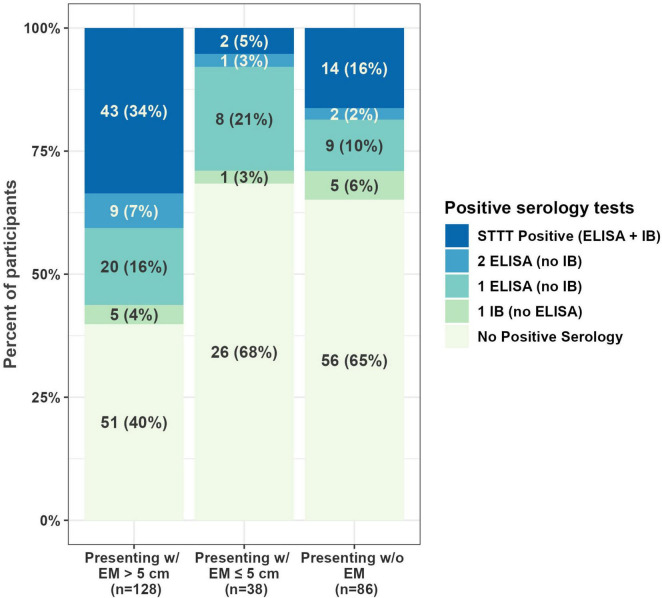
Serology results at first draw. One participant from WI (presenting with EM < 5 cm) provided a whole blood sample but was unable to provide a serology sample at the first draw, resulting in 252 samples with first draw serology results. EM, erythema migrans; STTT, standard two-tiered testing; ELISA, enzyme-linked immunosorbent assay; IB, immunoblot.

Serologic testing was performed on all samples. More than half (133) of first draw samples were negative on all serologic tests, and 24% (60) were positive on one tier only (Figure 1). Twenty-three percent of first draw samples (59) were STTT positive (Table 3). When stratified by single or multiple EM, 20% (28 of 140) enrolled with a single EM were STTT positive compared to 63% (17 of 27) of those enrolled with multiple EM (*p* < 0.001, one-tailed two-proportion *z*-test).

**TABLE 3:** Serology results.

STTT status (first draw)	East Hampton *N* = 123	Wisconsin *N* = 129	Total *N* = 252
Total STTT positive (IgM and/or IgG positive)	30 (24)	29 (22)	59 (23)
STTT positive (IgM positive alone)	23 (19)	18 (14)	41 (16)
STTT positive (IgG positive alone)	4 (3)	0 (0)	4 (2)
STTT positive (IgM and IgG positive)	3 (2)	11 (9)	14 (6)
**IgG Status**			
IgG negative on first draw	115 (93)	118 (92)	233 (92)
IgG newly positive on second draw	6 (5)	4 (3)	10 (4)

STTT, standard two-tiered testing. One participant from WI provided a whole blood sample but was unable to provide a serology sample at the first draw, resulting in 252 samples with first draw serology results.

When evaluating seroconversion, 10 of 233 samples (4%) that were IgG immunoblot negative on the first draw were IgG immunoblot positive at the second draw (Table 3). These 10 samples included 4 that were STTT positive by IgM but not IgG on the first draw and 6 that were STTT negative at the first draw, including 1 of the 6 that was PCR positive. Of the 10 samples demonstrating seroconversion, 6 were enrolled with EM > 5 cm, 1 was enrolled with EM < 5 cm, and 3 were enrolled without EM; 9 of the 10 were prescribed antibiotics by their provider for LD at enrollment.

First draw whole blood samples were tested by real-time PCR (RT-PCR) for *Borrelia* and other tick-borne pathogens (e.g., *Anaplasma*, *Babesia*, and *Ehrlichia*). Three samples, all from EH, were positive for *Borrelia* (2 *B. burgdorferi*, 1 *B. miyamotoi)*, 8 were positive for *Babesia microti* (7 EH, 1 WI), 8 were positive for *Anaplasma phagocytophilum* (1 EH, 7 WI), and 1 was positive for *Ehrlichia ewingii*/*canis* (EH). Approximately half of the samples positive for other tick-borne pathogens did not have laboratory evidence of LD, however 2 of the *A. phagocytophilum* samples from WI and 6 of the *B. microti* samples from EH were also classified as Laboratory Confirmed LD. The remaining 6 *A. phagocytophilum* samples, 2 *B. microti* samples, and 1 E. *ewingii*/*canis* sample were classified as SNL.

LDB samples were classified after blinded testing as previously described (23), with 30% (75) of first draw samples classified as Laboratory Confirmed LD (Table 4). Of the 75 samples classified as Laboratory Confirmed, 59 (79%) were enrolled with EM (56 with EM > 5 cm), and 16 (21%) were enrolled without EM (*p* < 0.001, one-tailed one-proportion *z*-test).

**TABLE 4:** LDB classification.

LDB classification	East Hampton *N* = 123	Wisconsin *N* = 130	Total *N* = 253
Laboratory Confirmed LD	41 (33)	34 (26)	75 (30)
Probable LD	43 (35)	29 (22)	72 (28)
Suspected LD	10 (8)	26 (20)	36 (14)
SNL (symptomatic no lesion)	29 (24)	41 (32)	70 (28)
**Method of confirmation for laboratory confirmed**	**East Hampton *N* = 41**	**Wisconsin *N* = 34**	**Total *N* = 75**
STTT positive	30 (73)	29 (85)	59 (79)
2 positive ELISA + EM > 5 cm	7 (17)	2 (6)	9 (12)
PCR positive	2 (5)	0 (0)	2 (3)
IgG seroconversion at second draw	2 (5)	3 (9)	5 (7)

Classification definitions: Laboratory Confirmed LD (positive STTT, 2 positive ELISAs with EM > 5 cm, positive PCR for *B. burgdorferi*, or IgG seroconversion); Probable LD (EM > 5 cm and STTT negative); Suspected LD (EM ≤5 cm and STTT negative), SNL (No EM and STTT negative). The denominator for method of confirmation is number of Laboratory Confirmed LD (EH = 41, WI = 34, Total = 75). LD, Lyme Disease; SNL, Symptomatic No Lesion; STTT, standard two-tiered testing.

### Symptoms at the convalescent draw

At the second draw, participants were asked “*Do you still have symptoms of LD?*”, and “*If yes, have you seen your provider about these symptoms?*” All participants were then asked “*Do you currently have any of the following symptoms*” about 13 specific symptoms common to LD. Twenty-two percent (17% EH and 26% WI) reported LD symptoms at the second draw. One quarter of those whose LD was confirmed by laboratory testing and 19% who enrolled with an EM > 5 cm whose LD was not confirmed by laboratory testing reported ongoing LD symptoms at the second draw (Table 5).

**TABLE 5:** Ongoing symptoms at second draw with antibiotic history.

	Laboratory confirmed LD (*n* = 75)	Probable LD (*n* = 72)	Suspected LD (*n* = 36)	SNL (*n* = 70)	Total (*n* = 253)
Reported LD symptoms at 2nd draw	19 (25)	14 (19)	3 (8)	19 (27)	55 (22)
Saw provider about ongoing symptoms*	8/19 (42)	4/14 (29)	1/3 (33)	6/19 (32)	19/55 (35)
**Antibiotic history**					
Prescribed oral antibiotics at enrollment	74 (99)	72 (100)	33 (92)	47 (67)	226 (89)
2nd Course oral antibiotics prescribed	3 (4)	0 (0)	0 (0)	2 (3)	5 (2)
3rd Course oral antibiotics prescribed	0 (0)	0 (0)	0 (0)	1 (1)	1 (< 1)

The denominator for Saw Provider About Symptoms is number of participants per category with LD symptoms at 2nd draw (Laboratory Confirmed LD = 19, Probable LD = 14, Suspected LD = 3, SNL = 19, Total = 55). LD, Lyme Disease; SNL, Symptomatic No Lesion.

For the 55 participants reporting ongoing LD symptoms (33 men and 22 women), the most common were joint pain (71%), fatigue (62%), and muscle pain (49%); other symptoms included headache (29%), night sweats (22%), flu like symptoms (22%), confusion/memory loss (18%), numbness/tremors (15%), gastrointestinal problems (13%), vision problems (11%), cardiac/respiratory problems (9%), hearing problems (4%), and facial paralysis (2%). These 55 participants had a median of 3 symptoms (mean 3.3), while the 198 reporting no ongoing LD symptoms had a median of 0 (mean 0.7) (*p* < 0.001, Wilcoxon signed rank test). Women reported more symptoms than men, median of 4 symptoms (mean 4.1) compared to a median of 2 symptoms (mean 2.7) (*p* = 0.016, Wilcoxon signed rank test). Despite the presence of ongoing symptoms of LD, only 35% (13 men and 6 women) saw their provider about their symptoms (Table 5).

### Antibiotic history

In this study, 89% of participants had been prescribed antibiotics for LD by their provider at enrollment or no earlier than 2 days prior to enrollment. Moreover, 21% (47/226) who were initially prescribed antibiotics reported ongoing LD symptoms ∼3 months later. Prescribing antibiotics at the initial visit was slightly more common at EH than WI (94% vs. 85%) (Table 5). Prescribing additional courses of antibiotics for LD between the first and second draws was rare; five participants received a second course and one of these received a third course of antibiotics.

## Discussion

LDB is a resource that provides well-characterized samples to investigators studying LD and other TBI, with more than 100 approved research projects to date. As part of LDB’s study design, participants enrolled with signs and symptoms of early LD were given the option to provide an additional sample and clinical information 2–3 months after enrollment. More than half of participants returned for the second draw. More men were enrolled at both sites, consistent with the higher incidence of early LD in men (24). Although more men than women reported ongoing symptoms of LD at the second draw and men were more likely to seek medical care for their ongoing symptoms, neither of these gender differences were statistically significant. However, women reported they experienced more symptoms than men, consistent with results from a large LD patient registry where, in patients clinically diagnosed with LD and remaining ill for 6 or more months after receiving antibiotic treatment, women experienced more severe symptoms and had worse functional impairment than men (25).

### Limitations of serology in early LD

This study’s findings, collected over a 10-year period, are well-aligned with previous studies demonstrating that STTT is insensitive in early LD. Only 23% of first draw samples and 34% of samples from participants enrolled with EM > 5 cm were positive by STTT. Further, first draw samples enrolled with multiple EM were more likely to be positive by STTT. One potential explanation for the low seropositivity rate is that the sites may have enrolled individuals before there was sufficient time for antibodies to form. For example, for the 115/167 participants enrolled with EM who reported the number of days they had the EM, the median was 4 days, mean 5.5 days, interquartile range 2–7 days, and maximum 21 days. The low seropositivity in early LD highlights the need for novel diagnostics to detect early infection. An additional finding of note is that for those enrolled without EM, STTT positivity was 16%, calling attention to the fact that individuals with LD can present without skin manifestations. Thus, healthcare professionals must maintain a high index of suspicion for early LD to improve timely diagnosis (7, 12, 26).

Results from this study demonstrate that IgG seroconversion is rare after antibiotic treatment. The 4% seroconversion rate reported here aligns with the findings from an earlier study of antibiotic treated EM patients that found that IgG seroconversion was infrequent (3/67; 4%) (27). Convalescent serologies following antibiotic treatment may not provide meaningful diagnostic information.

### Persistent symptoms in LD

LD has been associated with persistent symptoms in a subset of patients who received antibiotic treatment. Commonly described symptoms include fatigue, musculoskeletal pain, cognitive difficulties (28); headache and other symptoms have also been reported (21). Some patients experience persistent symptoms decades after the initial infection (21). The frequency of persistent symptoms after initial treatment in patients with LD varies across studies with estimates of up to 35% (21); however, 10–20% is frequently cited (20, 29). In this study, 21% (47/226) reported ongoing LD symptoms ∼3 months after initial antibiotic therapy. Similar to previous studies, joint pain, fatigue, muscle pain, and headache were common in this study’s participants. Moreover, these are the same symptoms that prompted participants to initially seek care. While symptoms related to memory problems were not asked at enrollment, confusion/memory loss was reported by 18% of participants at the second draw. In another study of well-characterized LD patients, neurocognitive difficulty was approximately 9% higher at 6 months than it was during the acute illness (30).

### Barriers to care

Of those reporting LD symptoms at the second draw, the majority (65%) did not see their provider about these symptoms. Thus, the prevalence of persistent symptoms after early LD treatment may be underestimated by providers. It is unclear why participants in this study who initially sought care for early LD did not see their provider when they were experiencing ongoing symptoms that they attributed to LD. It is well established that barriers to care exist in the US (31). Data from a large patient registry documented barriers specific to patients with persistent LD, including lack of insurance coverage, healthcare costs, travel time and distance to obtain care, and availability of care (32, 33). Barriers specific to early LD patients receiving care have not been adequately explored.

### Evidence for retreatment

Of the participants with ongoing symptoms who saw their provider, very few were prescribed additional courses of antibiotics between the first and second draws, yet there is evidence to support immediate re-treatment of patients with early LD. In a trial examining a 10–day doxycycline regimen in patients with EM, 7 of 22 were immediately retreated with an additional 10 days of oral antibiotics and another received ceftriaxone (34). In addition to Massarotti et al., investigators in six other US antibiotics trials of patients with EM, successfully immediately re-treated some participants who remained symptomatic or relapsed (14). One strategy for preventing ongoing symptoms of LD is for healthcare professionals to follow-up with their patients with early LD at the end of treatment to either verify the resolution of symptoms or to assess the need for immediate re-treatment in those patients who remain symptomatic, even if symptoms are mild (14). Additional research is needed to identify best practices to prevent ongoing symptoms in early LD patients and to predict which patients are more likely to experience these symptoms.

### Limitations

This study has several limitations. Enrollment criteria included participants presenting with or without an EM. It is possible that some of the skin lesions were not EM and some of the participants enrolled without EM did not have early LD. Additionally, only two geographic regions, Long Island and Central Wisconsin, were represented. Testing was performed at two different clinical labs that ran different ELISAs and immunoblots. Additionally, C6 peptide analysis was not commercially available for the entire study. This variability reflects laboratory testing in community settings, where clinicians have little control over the specific tests used in an STTT or MTTT protocol. It is possible that serologic results were influenced by variability in testing. Participant follow-up occurred at 3 months. Additional time points, such as 6 months or 1 year would provide more information about LD symptoms, and if symptoms reappeared or resolved. Finally, because symptoms of LD are non-specific and can be associated with other causes, it is possible that some symptoms experienced at the second draw were unrelated to LD.

## Conclusion

This 10 year prospective study of 253 participants in LD-endemic areas, conducted from 2014 to 2023, confirmed several earlier findings regarding early LD. With regard to serologic testing, this study confirmed that STTT is insensitive in early LD and that IgG seroconversion is rare following antibiotic treatment. With respect to therapeutic outcomes, this study confirmed that while the majority of people who receive antibiotics early in their disease do not report ongoing symptoms of LD, a significant number do. In this study 21% of participants continued to report symptoms of LD ∼3 months after antibiotic treatment. Healthcare professionals treating patients with early LD are encouraged to follow-up with their patients, assess them for ongoing symptoms, and consider antibiotic re-treatment as appropriate. Early diagnosis and treatment, with additional follow-up by healthcare providers, has the potential to improve patient outcomes, decrease the percentage of people who progress to persistent LD, and reduce the overall burden of LD.

## Data Availability

The raw data supporting the conclusions of this article will be made available by the authors, without undue reservation.
